# The principal components of electoral regimes: separating autocracies from pseudo-democracies

**DOI:** 10.1098/rsos.240262

**Published:** 2024-10-09

**Authors:** Karoline Wiesner, Samuel Bien, Matthew C. Wilson

**Affiliations:** ^1^ Institute of Physics and Astronomy, University of Potsdam, Potsdam, Germany; ^2^ Department of Political Science, University of South Carolina, Columbia, SC, USA

**Keywords:** democracy, applied statistics, political science

## Abstract

A critical issue for society today is the emergence and decline of democracy worldwide. It is unclear, however, how democratic features, such as elections and civil liberties, influence this change. Democracy indices, which are the standard tool to study this question, are based on the *a priori* assumption that improvement in any individual feature strengthens democracy overall. We show that this assumption does not always hold. We use the V-Dem dataset for a quantitative study of electoral regimes worldwide during the twentieth century. We find a so-far overlooked trade-off between election capability and civil liberties. In particular, we identify a threshold in the democratization process at which the correlation between election capability and civil liberties flips from negative to positive. Below this threshold, we can thus clearly separate two kinds of non-democratic regimes: autocracies that govern through tightly controlled elections and regimes in which citizens are free but under less certainty—a distinction that existing democracy indices cannot make.

## 1. Introduction

By the end of the twentieth century, scholars and practitioners alike had come to see democracy not only as universally valuable but also as a logical conclusion of political development [1,2]. However, two trends quickly sobered those optimistic views. The first was the observation of autocratic regimes with seemingly democratic institutional features, which some labelled ‘competitive authoritarian’ regimes or ‘illiberal democracies’ [3,4].^1^ The second was the potential for backsliding among established democracies, for which scholars lack explanations [5,6]. Countries such as the United States, India, Nicaragua, Poland and Hungary are often-cited examples of possible backsliders whose decline became apparent after 2015 [7–9]. Both trends evidence institutional changes that do not represent wholesale ‘democratization’ or ‘autocratization’ and that may not be clear from continuous measures of democracy that aim to characterize a country’s overall democraticness.

Early empirical treatments of democracy tended to represent it in a binary fashion (e.g. Schumpeter [10] and Huntington [11]), classifying countries as either democratic or not.^2^ However, the 1990s saw greater efforts to quantify democracy and the widespread use of continuous measures. Improvements in data collection and aggregation methods led to indices that combined together scores representing features such as the extent of competitiveness and inclusion and additional freedoms (e.g. [15–17]; for a review, see [18]).

Two of the most widely used democracy indices are the Polity IV democracy index [19] and the electoral democracy index (EDI) from the Varieties of Democracy (V-Dem) project. Like other examples, such as Alexander *et al.* [20] and Pemstein *et al.* [21], they measure multiple attributes and draw on different sources but tend to aggregate them into a single dimension that corresponds to being ‘more’ versus ‘less’ democratic. Although some applications have argued that democracy is best represented as two dimensions, such as by differentiating them based on contestation and participation (see [22,23,24]), it is much more common for empirical work to base conclusions about democracy on single-dimension measures. Nowhere is this clearer than in the heavily debated question of whether ‘mid-range’ democracies are more prone to conflict [25], which is rooted in the idea that democracy is a continuous, single-dimensional construct.

Large-scale indices that incorporate different features implicitly make the assumption that they correspond together so that an observation with a higher value on the overarching index has subcomponent values that are, on the whole, greater than or equal to those of an observation with a lower score. Observations with higher index values score either higher on all the individual measures or some components outweigh deficiencies in others. The question remains, however, whether ‘all things work together for good’ and whether being stronger on some features over others matters for political development. This is central to criticisms that composite indices mask important variations in regimes [26] and that inconsistencies between different aspects shape countries’ prospects for democracy [27,28].

To understand how elections and civil liberties influence democratic development in combination, we examine the multidimensional dataset provided by V-Dem, an ambitious coding project of hundreds of variables relating to attributes of democracy. By applying principal component analysis (PCA) to the subset of the V-Dem data that relates to electoral qualities of democracy over the period 1900–2021, we identify a two-dimensional subspace that contains 77% of the variance of the data. The second dimension represents the so-far overlooked trade-off between electoral control and citizen freedom. This allows us to clearly separate electoral autocracies from countries in which citizens are free but which potentially deal with corruption and violence as a result of those freedoms—a distinction that existing democracy indices cannot make. Furthermore, by studying the variables constituting the first and the second components, we discover a threshold in the first component at which the correlation between election capability and civil liberties turns from negative to positive. Only once this threshold is crossed do election capability and civil liberties seem to work together to enhance democraticness. This has important implications for understanding authoritarian resilience and adaptation.

## 2. Methods

### 2.1. Data

We use the publicly available dataset on electoral democracy collected and published by the V-Dem project.^3^ V-Dem is a large-scale collaborative effort that uses expert coding to generate quantitative data on aspects of democracy [29] for over 190 countries—dating back decades to centuries, depending on the country—with annual resolution. The project involves surveying a large number of country experts and using a Bayesian measurement model to estimate latent values [30]. The surveys tend to ask respondents to rate the level of openness and strength of an institution, such as election intimidation or media censorship, on an ordinal scale (e.g. ‘low’, ‘intermediate’ and ‘high’). Based on the responses, the measurement model estimates reliability between respondents and generates continuous point estimates for each question. The project combines information on those attributes into mid-level indices that represent specific concepts, such as the freedom of expression and election capability, which are also combined into an index of democracy.

One of the primary indices that V-Dem publishes is the EDI, which aims to measure the combined institutional guarantees suggested by Dahl [27]. The index is based on quantitative information on over 40 variables relating to electoral democracy that represent freedom of expression, freedom of association, suffrage, clean elections and elected officials.^4^ We selected all the indicators related to four out of the five concept groups, omitting indicators related to elected officials (*v2x_elecoff*) because they either are binary or nominal or are not applicable to all democracies.^5^ The relevant data yield 12 296 data points (i.e. country–year observations) for the period 1900–2021. In the electronic supplementary material, we give further details on the construction of the EDI and list the questions given to the experts to construct each of the 24 variables that we used. Electronic supplementary material, figures S6 and S7, illustrate the sample coverage, showing that missingness largely results from countries that went more than 5 years without holding an election and that independent countries across all regions are represented in the post-World War II period.

### 2.2. Principal component analysis

To evaluate the extent to which there are underlying dimensions in the data, we use PCA. PCA is one of the most simple and robust techniques for dimensionality reduction. It is part of a family of statistical techniques for representing high-dimensional data on a lower-dimensional linear subspace with as little information loss as possible. Although techniques have been developed for handling categorical data in a similar way [31,32], PCA and related approaches are primarily applied to continuous data because they rely on the computation of means and variances. The variance retained in the lower-dimensional subspace is a measure of the information that is kept. This method is, therefore, appropriate for the point estimates provided by V-Dem.

Following standard procedure, we normalized each V-Dem variable (i.e. centred it to a mean of zero and rescaled it to a variance of one) prior to performing PCA. For better readability of the plots, we rescaled all principal components uniformly such that the first component has a maximum absolute value of one (i.e. its values are bounded by [−1, 1]) while preserving the mean of zero for all components. We further reoriented each component such that its strongest loading was positive.

## 3. Results and discussion

**Figure 1.**
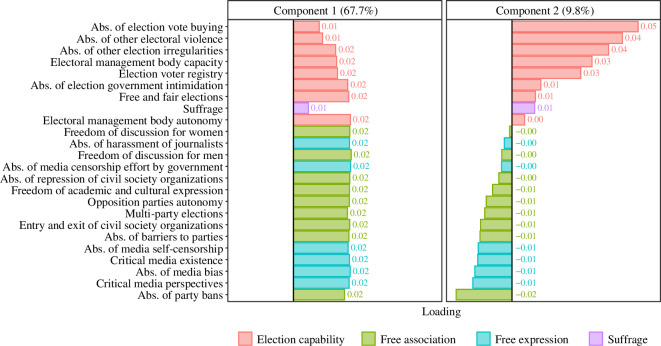
Variable loadings on first two principal components.

**Figure 2.**
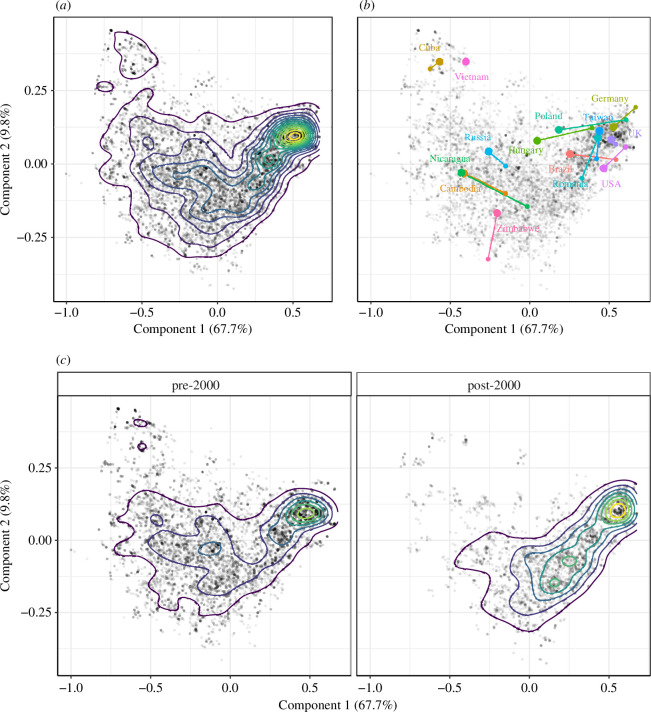
PC2 versus PC1 for all 12 296 data points (correlation =0 , per definition), (*a*) with kernel density estimation, (*b*) with example trajectories for the years 2011–2021 (small dot–large dot), and (*c*) separated into pre-2000 and post-2000 data, with kernel density estimation.

The main results of the PCA are summarized in figures 1 and 2. Figure 1 depicts how each V-Dem variable loads onto the first principal component (PC1) and second principal component (PC2). Shown in brackets is the variance that is retained by each component: together, the first two principal components account for almost 80% of variation in the data. Electronic supplementary material, figure S8, shows the cumulative variance retained as a function of the number of principal components that are included. Variables are colour-coded according to four classes: ‘free association’, ‘free expression’, ‘election capability’ and ‘suffrage’. The variable ‘suffrage’ is its own class and does not contribute significantly to either of the first two principal components. In fact, it predominantly loads onto the third principal component (explained variance: 4.8% and suffrage loading: 0.85; see electronic supplementary material, figure S8). This is possibly due to the fact that it is effectively a discrete variable with dominant values ( 0.5 for partial (e.g. male only) suffrage only and 1.0 for universal suffrage). In 2021, 98.8% of countries had universal suffrage.

The variance explained by the remaining 21 components ( ≈15% ) might be due to noise dominating in this regime, or it might indicate the existence of further patterns that have not been considered yet in existing measures, or even theories, of democracy.^6^ They might also indicate the existence of nonlinear correlations, which PCA would not be able to reveal.

By design, a high score in a V-Dem variable indicates ‘democraticness’ and a low score indicates the lack thereof (see §2.1 for details). As seen in figure 1, all V-Dem variables load positively onto PC1 (i.e. have positive correlations with it), with almost uniformly distributed weight. Hence, ranking countries according to their PC1 score, moving from most negative to most positive values, corresponds to ranking them in increasing democratic quality. The fact that the Pearson correlation between PC1 and EDI is very high ( ρ=0.941 ) shows that, overall, the EDI is well designed as a measure of democracy, insofar as the variables tend to move in the same direction.

PC2, on the other hand, has both positive and negative variable loadings. There is a clear distinction between variables relating to ‘free association’ and ‘free expression’, which all load negatively onto PC2, and variables relating to ‘election capability’ and ‘election management capacity’—specifically to the absence of vote buying, election irregularities and violence (i.e. all election variables except v2elmulpar)—which all load positively onto PC2. Thus, PC2 potentially represents a trade-off between variables associated with ‘election capability’^7^ (all of which have a positive weight) and variables representing ‘civil liberties’ (all of which have a negative weight). Mathematically, there are (at least) two ways in which a country can score intermediate on PC2 (the original variables take on negative as well as positive values): either it grants its citizens few civil liberties while maintaining a reasonable level of election capability, or it has a very high level of election capability while granting extensive civil liberties.^8^ There is only one way, mathematically speaking, in which a country can score high on PC2: it exhibits a very low level of civil liberties combined with a high level of election capability. We will revisit these cases in the discussion below (see figure 4).

The association between the first two principal components and the V-Dem variables suggests that PC1 more closely represents ‘democraticness’ associated with electoral competition combined with respect for civil rights and liberties, while PC2 indicates the trade-off between a state’s ability to effectively carry out elections and its respect for civil liberties. This is supported by the observation that some election variables that are particularly important for respecting citizens’ preferences, such as the autonomy of election management, election fairness and absence of intimidation, are more strongly associated with the first component. Notably, PC2 is very weakly correlated with the EDI ( ρ=0.124 ).

**Figure 3.**
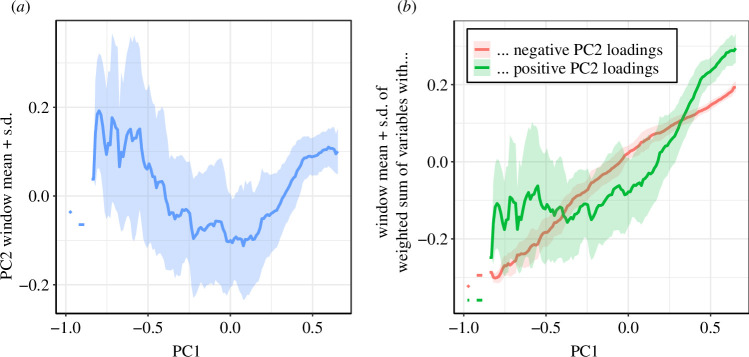
(*a*) The mean score of PC2 in a sliding window of PC1 with a width of 0.4 and a step size of 0.1. (*b*) The mean score of a decomposition of PC2—green curve: the weighted sum of variables relating to ‘election capability’ and ‘suffrage’, all of which positively correlated with PC2, and red curve: the weighted sum of variables relating to civil liberties (‘free association’ and ‘free expression’), all of which negatively correlated with PC2. Thus, the green curve minus the red curve equals the blue curve in (*a*) (see equations (3.1)–(3.3)).

In figure 2, all 12 296 country–year data points are plotted according to their PC1–PC2 values. Figure 2 *a* superimposes a standard kernel density estimate, which shows that the greatest concentration of observations is relatively high on PC1 (0.5) and somewhat lower on PC2 (2.4). Thus, the most populated area is more democratic and has moderately strong electoral control. At the same time, however, there is considerable spread in observations, with some scoring very low on PC1 and very high on PC2. It is suggestive that some regions of the PC1–PC2 plane are unpopulated. The reason will become clear in the discussion of figure 3 below. Figure 2*b* overlays example countries, showing that the upper-left area of the plot is occupied by electoral autocracies such as Cuba and Vietnam, which are characterized by weak civil liberties but that exercise tight control over elections as a tool for legitimation. Based on countries’ trajectories between 2011 and 2021, we also see that some more democratic regimes have moved away from the most populous area, which illustrates backsliding. The relationship between PC1 and PC2, therefore, reveals some important information. In figure 2*b*, we see that Cuba and Vietnam score higher on PC2 than, for example, Germany, Taiwan or the United Kingdom. The interpretation of the PC2 values will be clarified in the next section. In short, higher PC2 values do not indicate higher democratic quality. Instead, they indicate a trade-off between election capability and civil liberties, as can be seen from the variable loadings in figure 1.

### 3.1. Election capability versus civil liberties

To disentangle the two competing contributions to PC2, figure 3*a,b* graphs average values of PC2 against PC1, following a sliding window approach. In figure 3*a*, we plot the mean and standard deviation of PC2 against PC1 for all country–year events in a sliding window of width 0.4 and step size 0.1. As PC1 values increase, the average PC2 score first drops and then increases again, with a turning point around PC1 ≈0 . The variance in PC2 continuously decreases as one moves toward higher PC1 values, converging to a comparatively narrow distribution for PC1 values above ≈0.5 , while the average PC2 score reaches a plateau at around the same point.

To understand this nonlinear relationship between PC1 and PC2, in figure 3*b*, we separate the V-Dem variables into those that load negatively onto PC2 (red curve, variables relating to ‘free association’ and ‘free expression’ plus the variable v2elmulpar, which captures the presence of multi-party elections) and those that load positively onto it (green curve, variables relating to ‘election capability’, that is, all election variables except v2elmulpar).^9^ Mathematically, this corresponds to the following decomposition, using the values from the table in figure 1:


(3.1) $$\begin{array}{rl} {\text{PC2}}_{\text{pos.load}}= & \;0.5\cdot \text{‘Abs. of election vote buying` }+0.44\cdot \text{‘Abs. of other electoral violence`}\\ & +\cdots +0.05\cdot \text{Electoral management body autonomy}, \end{array}$$

(3.2) $$\begin{array}{rl} {\text{PC2}}_{\text{neg.load}}= & \;0.01\cdot \text{‘Freedom of discussion for women`}\\ & +0.03\cdot \text{‘Abs. of harassment of journalists`}\\ & +\cdots +0.22\cdot \text{‘Abs. of party bans`}, \end{array}$$

(3.3) $$\begin{array}{r} \text{PC2}={\text{PC2}}_{\text{pos.load}}-{\text{PC2}}_{\text{neg.load}}\;. \end{array}$$

Here, the dots are placeholders for the variables in between (see table in figure 1). In figure 3, these three aggregate values are plotted against PC1: the blue curve on the left hand side (PC2) is the sum of the green curve minus the red curve on the right hand side. Here, a clear pattern emerges. For low PC1 values (and, therefore, also low EDI values), the variables that account for ‘election capability’ (green curve, positive PC2 loadings) greatly fluctuate around an almost constant mean until a turning point around PC1 ≈ 0, above which the average of the ‘clean election’ variables continuously increases. The variables that account for ‘free association’ and ‘free expression’ show a very different behaviour: they continuously increase as PC1 increases, with very narrowly distributed values. The turning point in PC2 around PC1 ≈0 , which is clearly visible in figure 3*a*, is revealed as the point at which the quality of elections begins to rise above an apparently minimal value required for (semi-)democratic regimes. Thus, countries that score low on PC1 include non-democracies that exhibit well-coordinated elections but that do not allow civil liberties, while countries that score higher on PC1 tend to do both. The dip in PC2 that occurs as one moves from low to high values of PC1 indicates the electoral quality attributes being overshadowed by civil liberties, which illustrates a potential trade-off between election control and freedom that liberalizing regimes face. We interpret this dip as the point in democratic development at which feedback between election capability and civil liberties turns from negative to positive. This interpretation is supported by the fact that a significant part of the PC plane is empty. The latter indicates that a high value on PC1 is only obtained when all variables contribute high positive values. There are no cases of high PC1 values that aggregate very high values in election capability, which make up for very low values in civil liberties. Hence, in the high-PC1 part of the plane, ‘all things do work together for good’. In other words, in the region PC1 >0 , election capability and civil liberties are mutually enhanced, while in the region PC1 <0 , election capability and civil liberties can be mutually suppressed.

**Figure 4.**
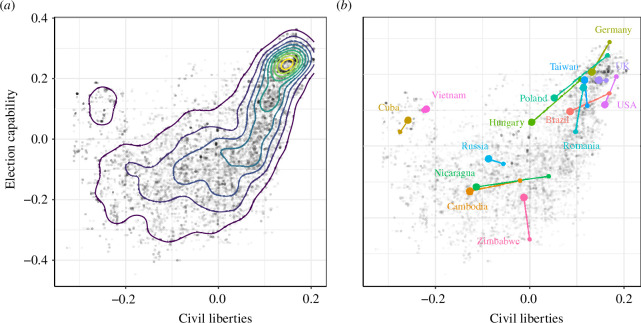
Aggregate positive versus negative PC2 loadings (i.e. ‘election capability’ versus ‘civil liberties’; see equations (3.1)–(3.3)) for all 12 296 data points, (*a*) with kernel density estimation and (*b*) with example trajectories for the years 2011–2021 (small dot–large dot).

In figure 4, we compare the two variable groups, with negative and positive loadings onto PC2, respectively, against each other. Labels of selected country positions in the years 2011 and 2021 are shown. The highest density of points—in the upper right—is that of countries with high levels of civil liberties and of election capability. These are generally considered to be democracies. In general, those countries with the greatest respect for civil liberties also have quality elections. As the level of control over elections decreases, civil liberties also decrease in general. At the lowest level of civil liberties (civil liberties values between −0.5 and −1.0 ), a broad range of values for ‘election capability’ are observed. The labels of selected country examples strongly suggest that countries with positive ‘election capability’ values and negative civil liberties values are electoral autocracies, which are known for holding elections but closely limiting opposition.

Overall, the data in figure 4 show that more democratic regimes are more likely to be able to effectively carry out elections but that some very non-democratic countries—which are quite low on the first component—are also associated with capable elections. This observation is well known in research on autocracies but has never before been demonstrated using continuous measures of democracy.

**Figure 5.**
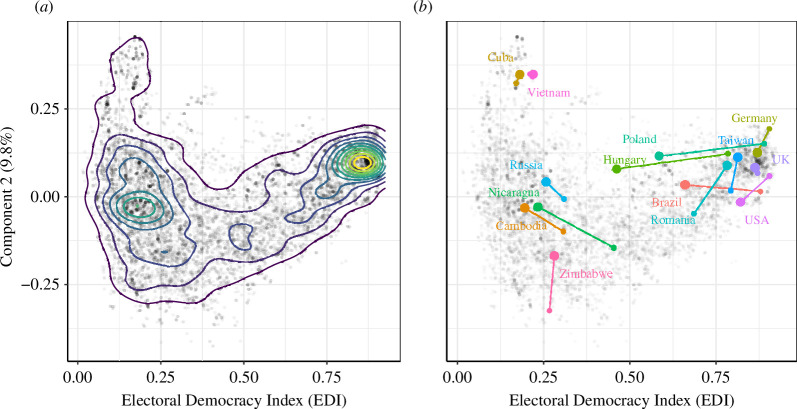
PC2 versus EDI for all 12 296 data points (correlation =0.124 ), (*a*) with kernel density estimate superimposed and (*b*) with examples for 2011–2021 (small dot–large dot).

Figure 5 plots countries’ PC2 versus EDI values. The resulting distribution is very similar to that in figure 2 (showing PC2 versus PC1) since EDI and PC1 are highly correlated ( ρ=0.941 ). Clearly visible is a high variance in PC2 for EDI values <0.5 . This indicates that differences between regime types among the non-democratic countries (such as electoral authoritarian and liberalizing regimes) are not resolved by the EDI but become visible in PC2. Importantly, countries that score high on the EDI tend to coalesce around similar values of PC2, but there is considerable variation at the lower end of the index that is associated with election capability. This is corroborated by specific examples of countries with EDI values around 0.25 —a group that includes electoral autocracies (Cuba and Vietnam), failed states (Cambodia and Zimbabwe) and countries such as Russia, which are considered to be in the process of solidifying an electoral authoritarian regime.

Comparing the change in EDI with the change in the positive and negative contributions to PC2 further reveals some interesting difference.^10^ One group of countries shows almost no change in their EDI value between 2011 and 2021, namely the United Kingdom, Taiwan and Zimbabwe (figure 5). However, they show a significant change in their positive and negative contribution to PC2: the United Kingdom has declined in its level of civil liberties, with the election capability remaining unchanged, while Taiwan and Zimbabwe have seen an increase in election capability while civil liberties have remained almost unchanged (on a high and low level, respectively; see figure 4). Other countries, such as the United States, have decreased significantly in all three values, as have Hungary and Poland.

This finding may help shed light on the quality of change captured in the term ‘democratic backsliding’, which has been applied to countries beset by greater political polarization and efforts by dominant parties to undermine the quality of elections under relatively free and fair regimes. To summarize, the nonlinear relationship between PC2 and democracy (EDI) suggests that—by exploring variation in the attributes thought to represent democracy—it picks up a potentially meaningful quality of political regimes and regime change that is obscured by the single dimension represented by the EDI.

## 4. Conclusions

Our results support three important conclusions. First, *the hidden dimension of democracy (PC2) uncovers an important distinction between countries with a similar democracy score (EDI and PC1) but differing levels of election capability and civil liberties*. Evaluating democratic improvements based solely on the EDI may, therefore, disguise changes in countries that are adopting a more stable form of autocracy by seeking to carry out controlled elections while also not respecting civil liberties. A good example of this is Zimbabwe, which saw a considerable increase in election capability and a decrease in civil liberties between 2011 and 2021 but did not show much change in its EDI score.

Second, *contrary to assumptions that election capability and civil liberties generally improve together, we identified a threshold in the first dimension at which the correlation between election capability and civil liberties flips from negative to positive*. Here, the interaction between these features of democracy appears to change from mutually enhancing to mutually suppressing. This finding suggests that there may be a trade-off between the order provided by *controlled* elections and the liberty that is associated with democracy. This also helps explain why some areas of the two-dimensional space tend not to be occupied.

Third, *the quality of elections is a stabilizing component of both democracies and (electoral) autocracies*. The many instances of countries with a high PC2 value and a low EDI (or PC1) value, which we identified as electoral autocracies, suggest that election capability might play a stabilizing role in electoral autocracies, despite the lack of civil liberties. However, it begs the question to what extent a decline in election capability has a destabilizing effect. Such an effect might be important for describing ‘backsliding’ among democracies, which we lack theories to explain [5]. The examples of Hungary, Poland and the United States (figures 4 and 5) suggest that the process of ‘democratic backsliding’ is accompanied (or even driven) by a greater decrease in election capability than in civil liberties. Notably, this differs from backsliding in other countries, such as Brazil and Nicaragua, which may represent different processes.

In view of these results, the question ‘which features strengthen or weaken democratic development?’ seems ill-posed. The appropriate question is perhaps, ‘how do features interact and, as a result, influence prospects of democratic development?’. The latter question captures more of the complexity of the concept of democracy [34,35].

## Data Availability

All data needed to evaluate the conclusions in the paper are present in the paper and/or publicly available online. The V-Dem dataset has been obtained from the publicly accessible source https://www.v-dem.net/data/the-v-dem-dataset/. The result of the PCA analysis is published on Dryad [36]. Supplementary material is available online [37].

## Footnotes

^1^
Examples include Azerbaijan, Zimbabwe, and Peru [3].

^2^
Many scholars still use a binary, categorical approach to representing democracy, such as Cheibub *et al*. [12] and Boix *et al*. [13], adhering to ‘minimal’ criteria to be considered democratic [14].

^3^
We use V-Dem Data v.12 (2022), https://www.V-Dem.net.

^4^
See ‘Appendix A: structure of aggregation’ in the V-Dem Codebook.

^5^
Examples of indicators that are not applicable to all democracies include measures about the relative powers of the head of government versus the head of state and differences between the lower and upper chambers in countries with bicameral legislatures.

^6^
We thank one of the reviewers for pointing this out.

^7^
We mean ‘election capability’ in the sense of ‘carrying out the elections effectively’ but not necessarily freely or fairly.

^8^
In fact, it is a continuous scale on which the election capability is matched by the lack of civil liberties.

^9^
A similar technique has been used successfully to identify scales and thresholds in Holocene social evolution [33].

^10^
It is worth keeping in mind that the relative contribution of PC2 to the variance of the data is 10%.
